# Spatially resolved metabolomics: From metabolite mapping to function visualising

**DOI:** 10.1002/ctm2.70031

**Published:** 2024-10-25

**Authors:** Xinyue Min, Yiran Zhao, Meng Yu, Wenchao Zhang, Xinyi Jiang, Kaijing Guo, Xiangyi Wang, Jianpeng Huang, Tong Li, Lixin Sun, Jiuming He

**Affiliations:** ^1^ School of Pharmacy Shenyang Pharmaceutical University Shenyang China; ^2^ State Key Laboratory of Bioactive Substance and Function of Natural Medicines, Institute of Materia Medica Chinese Academy of Medical Sciences and Peking Union Medical College Beijing China; ^3^ NMPA Key Laboratory of safety research and evaluation of Innovative Drug, Institute of Materia Medica Chinese Academy of Medical Sciences and Peking Union Medical College Beijing China

**Keywords:** clinical translation, drug discovery, mass spectrometry imaging, spatially resolved metabolomics, tumour metabolism

## Abstract

Mass spectrometry imaging (MSI)‐based spatially resolved metabolomics addresses the limitations inherent in traditional liquid chromatography‐tandem mass spectrometry (LC–MS)‐based metabolomics, particularly the loss of spatial context within heterogeneous tissues. MSI not only enhances our understanding of disease aetiology but also aids in the identification of biomarkers and the assessment of drug toxicity and therapeutic efficacy by converting invisible metabolites and biological networks into visually rendered image data. In this comprehensive review, we illuminate the key advancements in MSI‐driven spatially resolved metabolomics over the past few years. We first outline recent innovations in preprocessing methodologies and MSI instrumentation that improve the sensitivity and comprehensiveness of metabolite detection. We then delve into the progress made in functional visualization techniques, which enhance the precision of metabolite identification and annotation. Ultimately, we discuss the significant potential applications of spatially resolved metabolomics technology in translational medicine and drug development, offering new perspectives for future research and clinical translation.

**Highlights:**

- MSI‐driven spatial metabolomics preserves metabolite spatial information, enhancing disease analysis and biomarker discovery.
- Advances in MSI technology improve detection sensitivity and accuracy, expanding bioanalytical applications.
- Enhanced visualization techniques refine metabolite identification and spatial distribution analysis.
- Integration of MSI with AI promises to advance precision medicine and accelerate drug development.

## Graphical abstract

- The target samples underwent preprocessing and mass spectrometry imaging to achieve metabolite visualization. Through data analysis, functional visualization was accomplished, potentially leading to the discovery of biomarkers and therapeutic targets for clinical applications, thereby guiding precision medicine.

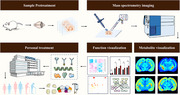

## 1 INTRODUCTION

As a powerful analytical tool, metabolomics technology plays an indispensable role in elucidating pathogenesis, drug effectiveness and targeted therapeutic strategies.^1^ Nevertheless, conventional liquid chromatography–tandem mass spectrometry (LC‒MS)‐based metabolomic approaches typically rely on sample homogenates for detection, resulting in the loss of spatial information pertinent to metabolites. With the development of mass spectrometry imaging (MSI) technology, spatially resolved metabolomics technology based on this imaging tool has received increasing attention as part of spatial multi‐omics,^2^ which can directly map metabolites from tissue sample sections and localise metabolites spatially. Furthermore, it provides a traceable pathway for elucidating the production and transformation of metabolites in vivo, as well as visualising the functional changes in the body induced by various drugs and diseases.

MSI technology has a diverse array of research applications, encompassing whole‐animal sections such as rats, mice and zebrafish,^3^ as well as insects such as bees.^4^ It is also employed in organ sections, such as those from the heart, liver, brain and spleen, as well as in tissue sections such as epithelial, bone and muscle tissues. High spatial‐resolution MSI offers remarkable capabilities, enabling imaging at the single‐cell level and even within tumour spheroids^5^ and organoids.^6^ Moreover, it has the same potential for plant sections to visualise the production and transformation of metabolites in vivo.^7^ This technique opens new avenues for metabolomics research, providing a powerful tool for exploring the complex relationships between metabolites and their environments at different scales.

**FIGURE 1:**
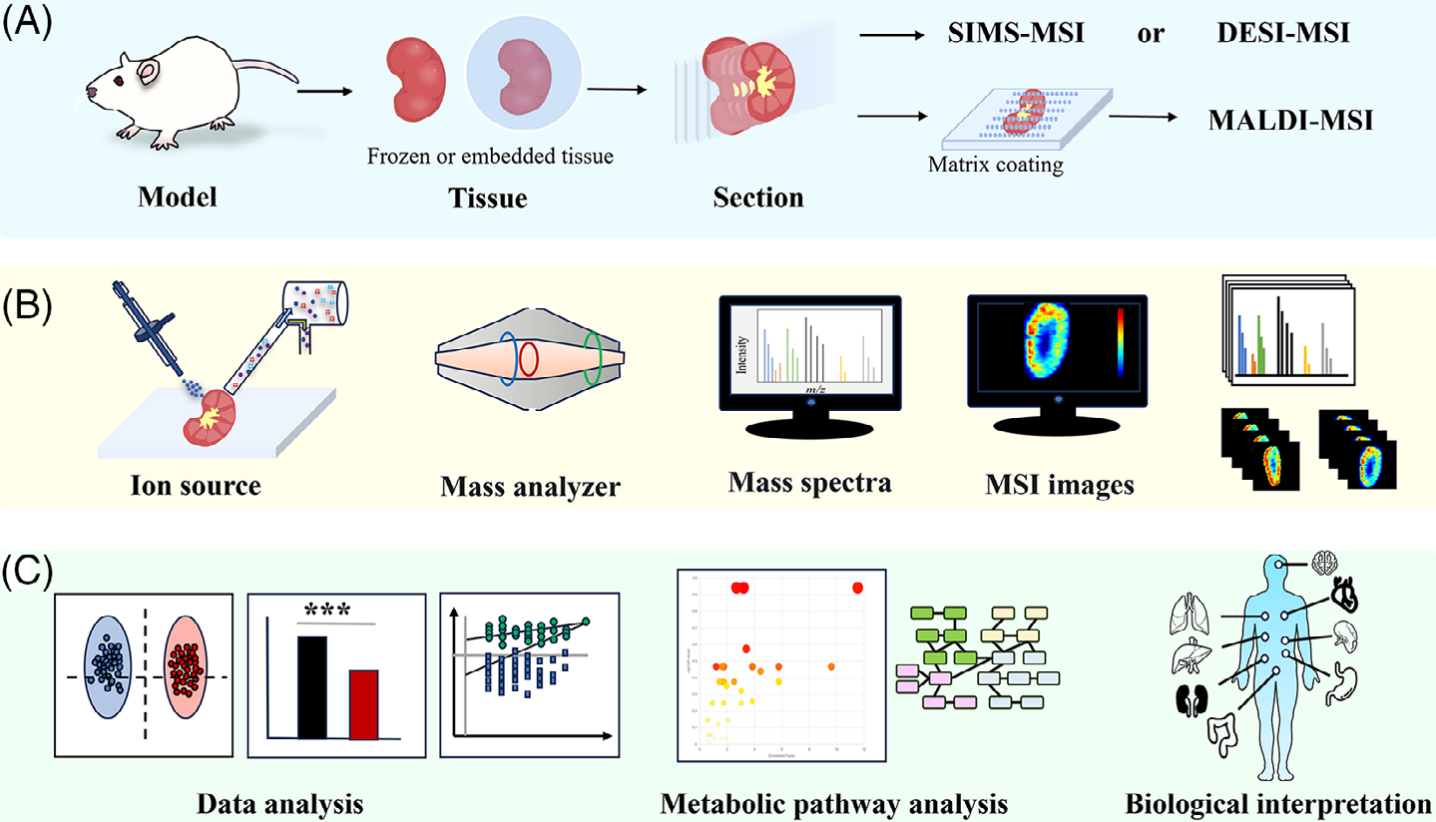
Typical workflow for spatially resolved metabolomics. (A) Sample acquisition and preprocessing. (B) MSI data acquisition and visualisation are achieved through desorption and ionisation by an ion source, followed by the acquisition of MS information via a mass analyser, resulting in numerous MSI images. (C) Data processing, metabolic pathway analysis and biological interpretation.

The spatially resolved metabolomics workflow encompasses several key steps: sample acquisition and preprocessing (Figure 1A), data acquisition with spatial information and image generation (Figure 1B), data processing and analysis and biological interpretation (Figure 1C). This comprehensive process ensures accurate and informative metabolite mapping, facilitating deeper insights into biological processes and functional changes in response to various exogenous stimuli and diseases.

## 2 ADVANCES IN METABOLITE MAPPING

### 2.1 Advances in sample pretreatment

Spatially resolved metabolomics technology enables label‐free, simple pretreatment, rapid and high‐throughput metabolite analysis, with widespread application in biomedicine. Nevertheless, ineffective sample ionisation results in weak signals and more noise, which constrains the specificity and sensitivity of metabolite molecule detection. Consequently, effective sample pretreatment is crucial for accurate analysis. The pretreatment process for spatially resolved histology samples (Figure 1A) typically involves sample collection, frozen section preparation and specific matrix coating in matrix‐assisted laser desorption ionisation (MALDI)–MSI.

High‐quality sections require a sound embedding strategy and section quality. Optimising a ‘standard operating procedure’ for MSI embedding enhances detection sensitivity, spatial resolution and reproducibility.^8^ In MALDI–MSI, embedding media such as optimal cutting temperature (OCT) compounds can inhibit ion formation and interfere with data analysis. To avoid this, mass spectrometry‐friendly embedding media such as carboxy methyl cellulose (CMC), polyacrylamide and gelatin are used instead of OCT. Unlike MALDI–MSI, desorption electrospray ionisation (DESI)–MSI does not require specific embedding media and can directly analyse fresh frozen tissue sections. Chen et al.^9^ reported that direct freezing and CMC embedding yielded comparable ion types in DESI–MSI, whereas OCT embedding resulted in lower total ion counts and signal intensities. Freshly frozen tissues provide greater spatial resolution and better biomolecular preservation, whereas formalin‐fixed paraffin‐embedded (FFPE) samples are more readily available in clinical settings and can be stored and transported at room temperature. The choice between fresh frozen and FFPE depends on the research goal. Wang et al.^10^ discovered a novel polyacrylamide gel embedding material that is superior to direct freezing and traditional media in MALDI–MSI, significantly enhancing metabolite ion signals and detection efficiency. Lenaerts et al.^11^ proposed a method for embedding organoids in the Epredia M‐1 cellulose matrix to preserve structural integrity.

The thickness of the sections affects the quality of the imaging. In MALDI–MSI, Liu et al.^12^ reported that a section thickness of 2−6 µm optimises brain tissue imaging, with thicker slices resulting in poorer outcomes due to the charging effect. Crouch et al.^13^ reported that a slice thickness of 10 µm resulted in the highest overall signal intensity compared with 12 and 14 µm thicknesses. Hamilton et al.^14^ developed 200 nm ultrathin cryosections to mitigate ice crystal damage. By optimising the embedding medium and slicing process, Stutts et al.^15^ improved the method for maintaining zebrafish tissue integrity. The embedding medium was a solution of 5% CMC and 10% porcine skin gelatin (G1890), which ensured successful sectioning. The samples were placed in a cryostat precooled to −20°C, and the light was kept off during slicing to prevent softening. Initial cuts were made at a thickness of 30 µm, followed by 16 µm thin sections. These optimisations addressed the challenges of adult zebrafish cryosectioning, supporting subsequent MSI analysis.

The matrix in MALDI enhances metabolite detection sensitivity and signal intensity through several mechanisms. They strongly absorb laser energy, promoting efficient desorption and ionisation of sample molecules. The matrix crystallises with analytes to form uniform crystals, improving ionisation efficiency. Some matrix act as proton donors, facilitating the protonation of analytes and enhancing positive ion detection. Additionally, the matrix protects analytes from laser‐induced damage, increasing their stability during analysis. A novel MALDI–MSI matrix, including 4‐nitrocatechol (4‐NC) for low‐molecular‐weight (LMW) compounds,^16^ Michler's ethylketone (MEK) for lipid imaging^17^ and 4‐aminocinnoline‐3‐carboxamide (4‐AC) for revealing metabolic abnormalities in Alzheimer's disease models,^18^ has been continuously discovered and applied.

The utilisation of novel derivatisation reagents and methods has facilitated the visualisation of challenging metabolites and led to advancements in compound‐specific mass spectrometry signal enhancement techniques, including on‐tissue chemical derivatisation, on‐tissue enzymatic digestion (OTED) and on‐tissue mass tag labelling strategies.^19^ For example, the application of 2‐fluoro‐1‐methylpyridinium p‐toluene sulfonate as a derivatisation reagent enhanced the ionisation efficiency of acetaminophen and related metabolites, enabling the detection of molecules with phenol moieties and low ionisation efficiencies.^20^ A combined chemical oxidation and derivatisation approach, coupled with air flow‐assisted desorption electrospray ionisation (AFADESI)–MSI, permitted the visualisation of hydroxyl‐containing metabolites in tissue sections.^21^ Additionally, the integration of Girard's P reagent with a hydrogel and α‐cyano‐4‐hydroxycinnamic acid significantly improved visualisation sensitivity.^22^ OTED has been employed for imaging tissues lacking histomorphology and gathering data for machine‐learning algorithms.^19^ Labelling strategies, such as the Phos‐tag‐based method by Aoki et al.,^23^ have enabled the visualisation of ‘invisible molecules’, such as low‐abundance lipids containing phosphate monoesters. Ye et al.^24^ introduced a stable isotope labelling MALDI probe to visualise endogenous thiols in tissues. Furthermore, Zare et al.^25^ proposed the use of boronic acid mass tags to label antibodies as MSI probes for visualising functional macromolecules in immunoassay‐based immuno‐DESI–MSI.

### 2.2 Advances in MSI data acquisition

**FIGURE 2:**
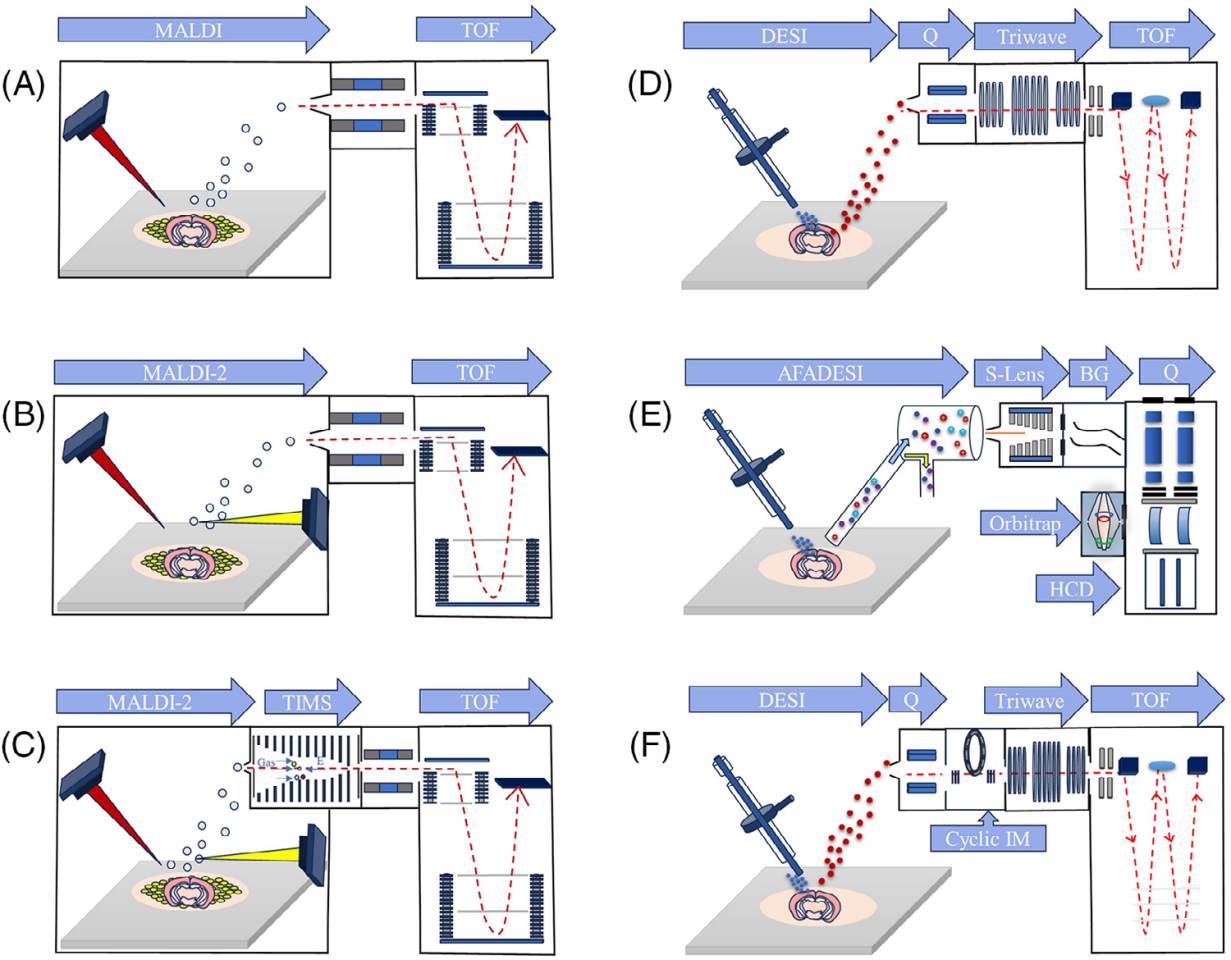
MSI instrumentation and platforms used for spatially resolved metabolomics. (A) MALDI–TOF; (B) MALDI‐2–TOF; (C) MALDI‐2–TIMS–TOF; (D) DESI–Q‐TOF; (E) AFADESI–Q‐Orbitrap; (F) DESI–Q‐Cyclic IM–TOF.

MSI data acquisition requires sample desorption and ionisation with position information from an ion source followed by the detection of ions with varying *m*/*z* values via a mass analyser (Figure 1B). MSI can be categorised into three types on the basis of the ion source: (1) secondary ion mass spectrometry (SIMS) imaging, which offers subcellular‐level spatial resolution and 3D imaging capabilities but is limited in obtaining comprehensive molecular information and is suitable for detecting fragment ions of biomolecules such as elements or metabolites; (2) MALDI–MSI, facilitated by matrix‐assisted laser desorption/ionisation, which boasts high resolution and is widely used in lipidomics, enabling the characterisation of macromolecules such as proteins and biopolymers, albeit with limited sensitivity for medium to high‐polarity metabolites; and (3) ambient ionisation represented by DESI–MSI, eliminates the need for complex sample pretreatment and matrix coating, boasts a broad metabolite detection range and preserves complete molecular information. Unlike the first two types, which require a vacuum environment, DESI can be operated at atmospheric pressure, although the spatial resolution is relatively rough. MALDI–MSI and DESI–MSI, the two most common technologies for spatially resolved metabolomics, have evolved into more mature instruments and platforms (Figure 2). MALDI can be connected with a variety of time‐of‐flight (TOF) analysers in commercial MSI platforms, such as Bruker's MALDI–TOF; DESI and AFADESI can be coupled with a variety of mass analysers; Waters has a commercial DESI platform; and many laboratories have constructed DESI platforms for quadrupole TOF (Q‐TOF), Orbitrap and triple quadrupole (QqQ). AFADESI has been developed into a highly advanced commercial ion source that can be used in conjunction with various mass spectrometry analysers with atmospheric pressure interfaces, such as Orbitrap, Q‐TOF and QqQ. Furthermore, ion mobility technology (IM), such as trapped ion mobility spectrometry (TIMS) from Bruker and cyclic IM from Waters, can be employed in conjunction with anion source to improve separation and imaging.

#### 2.2.1 SIMS

SIMS–MSI technology uses an ion beam to bombard the surface of a sample to generate secondary ions, which are examined by high‐resolution mass spectrometry (HRMS), enabling the investigation of the chemical composition of biological samples with high spatial resolution and high mass resolution. SIMS achieves 3D imaging by sequentially ablating sample layers, typically using an ion beam to remove surface material layer‐by‐layer, resulting in a stack of 2D mass spectrometry images at varying depths. These 2D images are then compiled to produce a three‐dimensional representation of the elemental or molecular distribution within the sample. Owing to its high spatial resolution at the nanometre scale, SIMS provides extremely fine depth resolution, making it particularly suitable for analysing nanoscale structures on sample surfaces. However, the depth profiling process can cause sample damage, potentially affecting subsequent analyses. Additionally, while SIMS excels in mapping the 3D distribution of elements and small molecules, it faces challenges when analysing larger or more complex organic molecules.

In 2017, Gilmore's team^26^ reported that 3D SIMS Orbitrap can achieve metabolic imaging at subcellular resolution, and by combining 3D Orbitrap with TOF‐SIMS, intact sulfatide and phosphoinositol lipids were imaged with a spatial resolution of less than 2 µm, and a mass resolution of greater than 480 000 at *m*/*z* 200 was achieved. Scurr et al.^27^ annotated 16 proteins (up to 272 kDa) through head peptide sequencing via protein ballistic fragmentation induced by a gas cluster ion beam (GCIB), which resulted in peptide cleavage and produced fragments for subsequent Orbitrap analysis.

Gilmore's team developed a Cryo‐Orbi–SIMS method, leveraging Orbitrap SIMS, to analyse bacterial biofilms in their frozen hydrated state, enhancing polar molecule signals by approximately 10 000‐fold and nearly doubling the number of detected molecules. Tyler et al.^29^ employed Cryo‐TOF–SIMS to capture 3D images of ciprofloxacin in biofilms, achieving cellular‐level resolution. Benkovi et al.^30^ conducted in situ 3D submicron chemical imaging of single cells via GCIB–SIMS to visualise purine biosynthesis directly from the ground up via the multi‐enzymatic complex purinosome. Sjövall et al.^31^ used TOF–SIMS for 3D molecular imaging of the stratum corneum, revealing the spatial distribution of lipid molecules and the heterogeneous distribution of oleic acid cholesterol, providing insights into dermal medication penetration mechanisms.

#### 2.2.2 MALDI

MALDI–MSI offers an optimal balance between sample preparation efficiency, chemical sensitivity and spatial resolution, making it one of the most widely utilised ionisation techniques. The process begins with the application of a LMW matrix to the tissue sample. Subsequently, a laser beam is directed onto the crystalline matrix, transitioning it from a solid to a gaseous state. This transition facilitates ionisation of the sample, which is then analysed through MSI to detect the ions and produce detailed metabolite images. MALDI–MSI, DESI–MSI and AFADESI are also capable of performing 3D MSI, but on a different technical principle than SIMS, which focuses on slicing the sample and performing 2D mass spectrometric imaging on each slice. The resulting 2D image is then reconstructed to produce a 3D image of the sample. This method also has its limitations, including relatively low spatial resolution (in the micrometre range), the need for a large number of slices, a time‐consuming process and complex sample preparation. The depth resolution of the 3D image is determined by the thickness of each slice.3D imaging by MALDI–MSI is particularly beneficial for analysing the spatial distribution of large biomolecules such as proteins and peptides in a sample, providing valuable insights for histological studies. MALDI is widely used for analysing large molecules such as biopolymers, and the continuous discovery of the MALDI matrix has significantly broadened metabolite coverage. Luo et al.^32^ achieved enhanced sensitivity and simplicity in cholesterol species visualisation by utilising methylpyridinium formaldehyde for reactions with cholesterol and hydroxyl‐containing sterols. Wang et al.^16^ introduced 4‐NC to boost the imaging of LMW compounds and MEK for lipid imaging enhancement^17^ and recently screened 4‐aminoazobenzene as a novel negative‐ion MALDI matrix,^33^ improving the in situ detection and imaging capabilities of MALDI–MSI for metabolites. Zhao et al.^34^ employed polyvinylpyrrolidone‐capped silver nanoparticles as a matrix for concurrent analysis and imaging of diverse lipid classes.

Additionally, various advancements in MALDI techniques have been reported. Guo et al.^35^ achieved concurrent molecular imaging of phospholipids and low‐abundance free fatty acids in thyroid cancer tissue sections through tissue derivatisation. Hummon et al.^36^ visualised and quantified d10‐irinotecan in individual cell spheres via an internal standard. Islam et al.^37^ demonstrated that AP‐MALDI–MSI can rapidly localise small molecule drugs and their metabolites at speeds of up to 32 pixels/s. Setou et al.^38^ visualised the distribution of acetaminophen (APAP) and its metabolite APAP–CYS in the kidney at different time points with high spatial resolution via AP‐MALDI.

Typically, ultraviolet lasers are used for MALDI. However, infrared lasers MALDI (IR‐MALDESI), which involves ionisation mechanisms distinct from those of conventional MALDI, have shown significant promise in various applications. Segura et al.^39^ performed the first spatial investigation of brain N‐linked glycans via IR‐MALDESI–MSI. This technology has resulted in a substantial increase in N‐linked glycan detection. Heiles et al.^40^ boosted signals by introducing an in‐capillary dielectric barrier discharge module to the post‐ionisation of neutrals from the IR‐MALDI–MSI source, achieving lateral resolutions of 20 µm. Furthermore, IR‐MALDESI–MSI enables whole‐body MSI of zebrafish, covering a broad range of lipids at high spatial resolution.^15^

#### 2.2.3 MALDI‐2

Laser‐induced post‐ionisation (MALDI‐2) is a secondary laser ionisation method based on a MALDI ion source. It utilises an additional laser to further ionise the neutral species generated in the initial MALDI process, thereby allowing for improved ionisation efficiency and increased sensitivity. Dreisewerd's team^41^ developed a t‐MALDI‐2 ion source by combining transmission‐mode MALDI–MSI (t‐MALDI–MSI) with MALDI‐2. This, along with the Orbitrap mass analyser, addresses the reduced ion abundance in MALDI at small pixel sizes, enabling brain tissue analysis at a 600 nm pixel size.^41^ McKinnon et al.^42^ applied negative ion mode MALDI–MSI with MALDI‐2 to MSI of small metabolites, revealing the superiority of MALDI‐2 in terms of metabolite coverage and sensitivity. MALDI‐2 achieves a 20 µm pixel size in imaging mouse liver tissue with metastatic mammary carcinomas, detecting six tumour‐specific metabolites undetectable by conventional MALDI and providing up to 20‐fold higher signal intensity for metabolites such as glutamate.^42^ Heijs et al.^43^ coupled MALDI‐2 to a trapped IM TOF mass spectrometer, enhancing the analytical sensitivity of N‐linked glycans by boosting N‐glycan molecular [M−H]^−^ species by approximately 3 orders of magnitude and [M+Na]^+^ adducts by approximately 10‐fold. Heeren et al.^44^ demonstrated the first analysis of synthetic polymers via MALDI‐2, which improved the sensitivity across all the polymers tested, highlighting its potential for studying the distribution of certain classes of polymers in biological systems.

Recently, Pan et al.^45^ developed a high spatial resolution transmission atmospheric pressure laser desorption ionisation technique combined with a compact post‐photoionisation (t‐AP‐LDI/PI) MSI technique. Like MALDI‐2, this technique uses laser desorption and subsequent ionisation to improve detection sensitivity. They successfully applied this method to the imaging analysis of mouse brain cerebellum and melanoma tissues, visualising the spatial distribution of various metabolites and lipids. Additionally, by optimising experimental parameters such as the laser power, frequency and scanning speed, this method can achieve a high spatial resolution of 4 µm, which is conducive to single‐cell‐level imaging analysis of tissue samples.^45^

#### 2.2.4 DESI

DESI is a powerful ambient ionisation technique that facilitates the direct analysis of biological samples, such as tissue sections, without necessitating extensive sample preparation.^46^, ^47^ By utilising a stream of charged droplets to interact with the sample surface, DESI effectively desorbs and ionises molecules, making them available for mass spectrometric analysis. A primary advantage of DESI is its ability to ionise samples under atmospheric pressure, thus eliminating the need for a vacuum chamber, which is typically required in SIMS or MALDI. Consequently, DESI–MSI can be analysed directly on the sample surface without the need for complex sample pretreatment steps, such as SIMS–MSI for enhanced conductivity through metal coating or MALDI–MSI for enhanced ionisation efficiency and mass spectrometry signal intensity through deposition of the matrix. The principle of 3D imaging with DESI–MSI is the same as that of MALDI–MSI, where a 3D image is generated by scanning different layers of the sample and reconstructing the data into a 3D image, however, a significant advantage of DESI–MSI is the ability to simplify sample preparation. Together with the different ionisation modalities, DESI is more suitable for detecting the 3D distribution of small molecules and pharmaceutical compounds, especially in complex biological tissues. DESI is especially well suited for analysing LMW polar compounds, as it minimises fragmentation, thereby allowing for the direct detection of these compounds. DESI is a versatile and powerful technique for the analysis of biological samples, offering unique advantages in simplicity, sensitivity and imaging capabilities.^48^ This makes it an ideal technique for metabolite mapping and, therefore, spatially resolved metabolomics analysis. Nevertheless, a significant limitation of DESI lies in the electrospray process, which can compromise spatial resolution. Researchers continue to work on addressing this issue and improving the performance of DESI for high‐resolution imaging and spatially resolved metabolomics.

Pan's group^49^ developed DESI‐PI‐MSI, a technique that integrates post‐PI to increase the detection of non‐polar molecules and lipid sensitivity. The ammonia‐assisted DESI/PI MSI approach significantly increased the signal intensity of endogenous metabolites in mouse brain tissue by 37.1‐fold.^50^ Nano‐DESI, a room‐temperature ionisation technique, generates multiple charged protein ions for protein analysis. Laskin's team^51^ employed nano‐DESI to image uterine tissue sections with spatial resolutions below 10 µm, achieving resolutions as fine as 7 µm through optimised capillary probes and section thickness.^52^ They also demonstrated the spatial localisation of N‐linked glycans in biological tissues via nano‐DESI–MSI.^53^ Coupled with a triple quadrupole mass spectrometer and multiple reaction monitoring, nano‐DESI enables the differentiation of isobaric phospholipids, which typically require a mass resolution of 3.8 million, and facilitates the imaging of arachidonic acid‐like isomers, establishing a cost‐effective platform.^54^

#### 2.2.5 AFADESI

In 2011, our team pioneered the development of AFADESI–MSI technology, building upon the foundation of DESI.^55^ Like DESI–MSI, AFADESI–MSI also employs ambient ionisation but has enhanced ionisation efficiency and molecular detection capabilities and is particularly suitable for large‐sample 3D imaging. This innovative technique uses a high extraction airflow through an extended transfer tube, optimising the desorption of charged droplets, enhancing ion collection and improving the ion transfer efficiency to achieve unparalleled sensitivity. AFADESI technology enhances sensitivity through the optimisation of ion collection, transmission and desolvation processes via high‐speed airflow, as shown in Figure 2E. This results in a more compact ion packet that is more than 75 times more sensitive than traditional DESI ionisation methods.^56^ In addition, the modular design of the AFADESI system makes it compatible with most commercially available mass spectrometers, extending its applicability and range.

When coupled with the Q‐Orbitrap, AFADESI technology has been shown to exhibit a wide dynamic range, enabling the imaging and analysis of over 1500 metabolites.^57^ This results in comprehensive mapping of metabolite species within biological tissues, providing valuable insights into cellular processes and disease mechanisms. The combination of AFADESI and Q‐Orbitrap offers a powerful and versatile tool for metabolomics research,^58^ with the potential to uncover novel biomarkers and pathways in complex biological systems.

AFADESI–MSI uses a unique ion transport tube design that overcomes the spatial coverage and sensitivity limitations of other MSI technologies in whole‐body imaging. This design efficiently collects and delivers charged droplets containing desorbed samples, enhances interactions with air molecules, increases solvent evaporation and ion formation efficiency and minimises ion loss. In contrast, while MALDI and SIMS are effective for small areas, they struggle to maintain high sensitivity and resolution over larger tissue sections owing to ion transport and sample desorption limitations. The innovative ion transport technology of AFADESI–MSI enables comprehensive and detailed whole‐body imaging. With the design and switching of the newly developed fine probe (P‐100) and large probe (P‐200), it is possible to image both microregions of brain tissues at high resolution and whole animal sections.^58^ It has been employed to investigate drug distribution and metabolic differences in rats for the sedative‐hypnotic drug candidates YZG‐331 and YZG‐330.^59^ Additionally, whole‐body analysis of zebrafish via AFADESI–MSI has provided rich functional metabolite information for more than 1000 metabolites, revealing organ‐specific metabolites in nine regions. Integrated with metabolic pathway analysis, this technique has mapped the first global metabolic network with zebrafish in situ data, facilitating the elucidation of human disease mechanisms through zebrafish models.^3^

AFADESI–MSI also has the ability to discern organ microregions. By imaging the metabolite distribution in brain microregions and focusing on polar small molecules with *m*/*z* 50−500, high‐resolution visualisations of representative metabolites such as γ‐aminobutyric acid, adenosine, acetylcholine and fatty acids were achieved, leading to the mapping of a metabolic network encompassing 20 metabolic pathways in the rat brain.^60^ AFADESI–MSI was employed to visualise metabolites within 3D tumour‐immune cell coculture spheroids, revealing significant alterations in glutamine catabolism and related metabolic enzymes.^61^ Recently, Wan's team^62^ capitalised on AFADESI–Orbitrap–MSI to spatially resolve the toxicokinetic profile of multiple organic pollutants in zebrafish. This marked the first successful MSI of complex polyhalogenated compounds, underscoring the potential of AFADESI in environmental science.^62^

## 3 ADVANCES IN FUNCTION VISUALISATION

The development of MSI‐based spatially resolved metabolomics requires more accurate annotation of metabolites in addition to coverage of more metabolite types, and more complex causal analysis of metabolites by various means for functional visualisation and biological interpretation (Figure 1C,D).

### 3.1 Advances in metabolite visualisation and annotation

Owing to the absence of chromatographic separation in MSI, differentiating between molecules sharing the same *m*/*z* values, such as isomers and enantiomers, becomes challenging. HRMS analysis for accurate mass measurement,^54^ MS/MS data or strategies such as derivatisation are required for proper identification. Fortunately, the incorporation of IM has facilitated the precise annotation of isomers by acquiring their molecular collision cross‐sections from IM analyses.

Ouyang et al.^63^ utilised Waters Cyclic IM and DESI for rapid ion focusing and separation, allowing the distinction and localisation of lipid isomers. IMS enabled quick separation of lipid ions from each pixel in 150−300 ms, facilitating fragmentation analysis of coeluting lipid ions. Different lipid ions were eluted at distinct times. By deconvolving mixed mass spectrometry signals via an algorithm, they reconstructed standard MS/MS spectra for each lipid. This method supported unbiased data‐independent acquisition mode lipid MS/MS imaging, thereby enhancing sample utilisation and analytical coverage. The imaging technique separated and identified over 40 structurally distinct lipids in mouse brain tissue and identified a potential cancer biomarker, the phosphatidylethanolamine (PE 18:1_18:1) isomer, in human hepatocellular carcinoma tissue. This approach significantly improved lipid separation and structural identification, offering new possibilities for spatial lipidomics research.^63^

Julia et al.^64^ developed a portable nano‐DESI–MSI device integrated with a drift tube IM spectrometer–mass spectrometer to separate isomers and tautomers via IM–MSI. The abundance of each isomer was manually extracted via peak‐fitting, and IM–MSI created ion images to separate the lipid isomers. IM–MSI effectively differentiates isomers such as sodium‐adducted PE (40:4) and PC (P‐38:3), which are indistinguishable by mass but resolved in IM mode. Additionally, IM–MSI minimises solvent‐induced background interference, as shown by separating the [M+Na]^+^ ion from LPC's isobaric solvent peak (18:2). Thus, combining nano‐DESI–MSI with IM shift separation enhances the molecular specificity of MSI investigations by separating isomeric species in complex biological samples.^64^ IM separation not only mitigates isotopic and isomeric interferences but also aids in obtaining precise concentration gradients in samples via MSI. Furthermore, the structural insights provided by IM separation are crucial in revealing the intricacies of biological systems.

LC‒MS‐assisted MSI identification further enhances this capability by providing chromatographic separation before mass spectrometric analysis, thereby improving the specificity of metabolite detection and characterisation. Although MSI technology has relatively low sensitivity, limiting its ability to detect most metabolites, it can still provide spatial distribution information related to the mechanism of drug action. Sun et al.^65^ combined UPLC–ESI–Q‐TOF–MS and MALDI–MSI techniques to conduct a comprehensive study on the changes in the content and spatial distribution of major flavonoid compounds in Tartary buckwheat fruit. Abliz et al.^66^ utilised spatial distribution information of neurotransmitters in brain tissue, provided by MSI techniques, alongside the results of neurotransmitter metabolites detected by LC–MS techniques. This approach was used to further elucidate the mechanism of the sedative‐hypnotic action of the herbal YZ extract.^66^ Tang et al.^67^ quickly separated and identified the major chemical components in P. quinquefolius via UPLC–Q‐TOF/MS. Combined with DESI–MSI analysis, most of the labelled compounds presented a specific spatial distribution and different relative amounts at different sites.^67^

**FIGURE 3:**
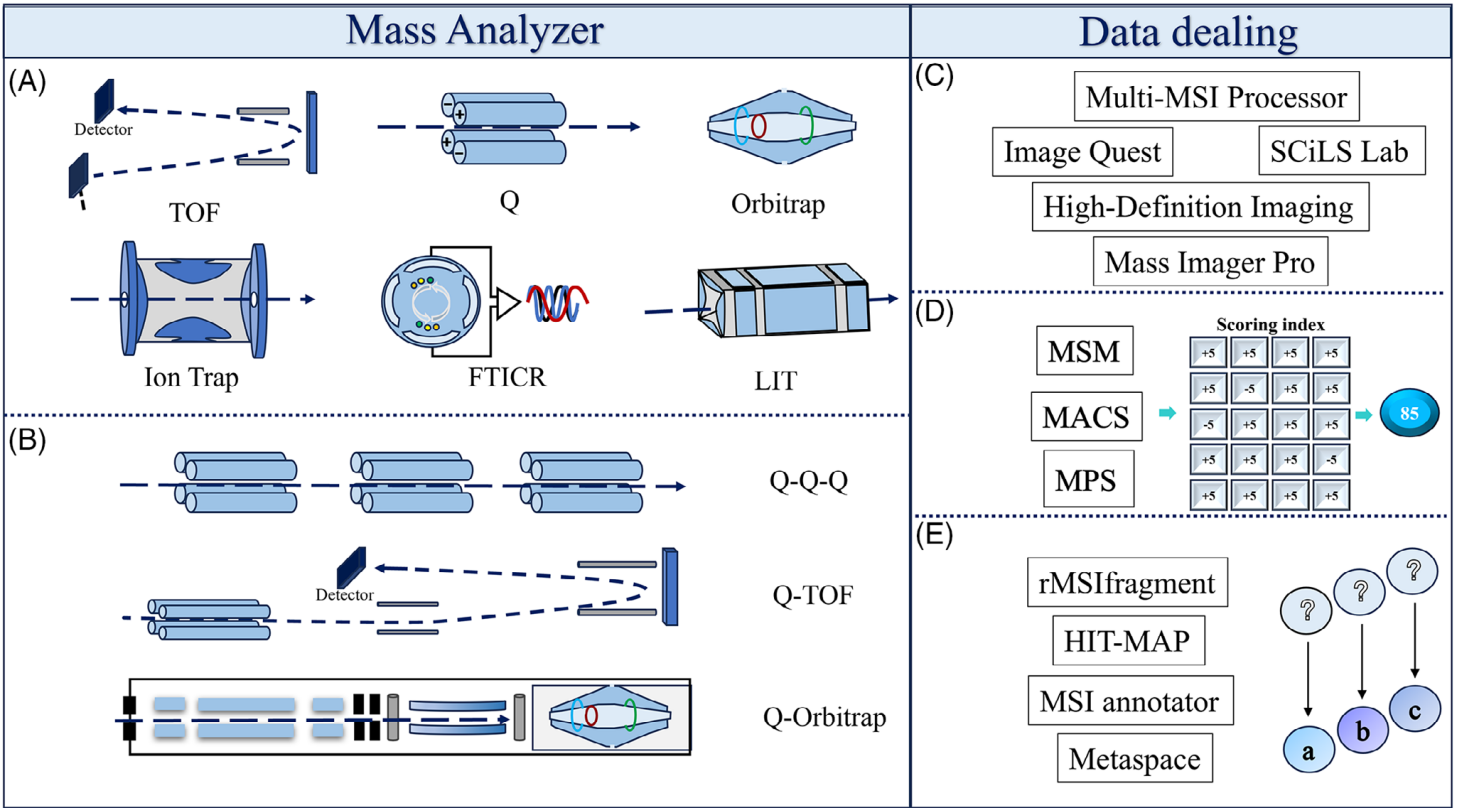
Comparison of different ions distinguished by mass analyser and annotation by data processing. (A) Mass analyser: TOF, quadrupole mass analyser (Q), Orbitrap mass analyser (Orbitrap), ion trap mass analyser (Ion Trap), Fourier transform ion cyclotron resonance (FTICR) and linear ion trap mass analyser (LIT). (B) Tandem mass analyser: triple quadrupole mass analyser (TQMS or QqQ), Q‐TOF, hybrid quadrupole Orbitrap mass spectrometer (Q‐Orbitrap). (C) Visualisation software: multi‐MSI processor (MMP),^68^ Image Quest (Thermo Fisher Scientific), SCiLS Lab (Bruker), high‐definition imaging (Waters), Massimager Pro (Chemmind Technologies). (D) Data scoring system: MSM (in the METASPACE platform), metabolite annotation confidence score (MACS),^69^ metabolic perturbation score (MPS).^70^ (E) Automatic annotation tool: rMSIfragment,^71^ HIT‐MAP,^72^ MSI annotator^73^ and METASPACE.^74^ ^.^

Accurate metabolite identification is essential for enhancing the efficacy and development of spatially resolved metabolomics. However, the complexity and high dimensionality of MSI data pose challenges in extracting bioinformatically relevant features from large datasets and conducting spatially resolved metabolomic investigations. The advancements in MSI technology, with enhanced sensitivity and resolution, have led to increases in both the quantity and diversity of detected metabolite data, rendering simple screening and database matching insufficient. Consequently, metabolite annotation is evolving to embrace intelligence and automation approaches, going beyond reliance solely on mass spectrometry for compound recognition (Figure 3).

For example, the progress of histochemical derivatisation has generated extensive and intricate datasets, expanding compound coverage. Lee et al.^75^ introduced a pipeline utilising METASPACE for rigorous annotation of derivatised MSI tissue data. Compared with manual processing, this method reduces processing time, provides systematic scoring and annotation, and eliminates 5−25% of erroneous derivatisation annotations.^75^ High‐resolution accurate mass (HRAM) imaging datasets are annotated via the METASPACE platform, using metabolite signal matching (MSM) scores as a confidence measure. Nevertheless, MSM scores can be inconsistent and may not reflect accurate confidence levels. The metabolite annotation confidence score (MACS), a novel MSI identification scoring tool, offers an alternative scoring system based on MSI metrics (mass measurement accuracy, spectral accuracy and spatial distribution). MACS accurately and reproducibly scores annotations, reflecting confidence levels in HRAM–MSI datasets.^69^

Recently, Baquer et al.^71^ presented rMSIfragment, an open‐source R package designed to enhance MALDI–MSI lipidomics through automated in‐source fragment annotation. This package annotates lipids via recognised adducts and fragmentation pathways, employing a new scoring system to increase annotation confidence and minimise false positives across various MALDI–MSI sample types and experimental setups.^71^ Additionally, our team introduced an organ specific, metabolite database‐driven approach for precise MSI metabolite annotation. We developed MSI Annotator, an automated tool that efficiently uncovers spatial metabolic shifts throughout the mouse body.^73^ Furthermore, Du et al.^68^ introduced multi‐MSI processor (MMP), a novel MSI data processing software that integrates data reading, MSI visualisation, post‐processing data preservation and biomarker discovery. While primarily focusing on the AFADESI–MSI platform, the MMP also supports other MSI platforms and enables swift MSI analysis workflows, assessing data quality, screening differential MS peaks and identifying abnormal metabolic pathways.^68^ Another significant research challenge lies in the use of MSI for simultaneous metabolite quantification and characterisation. To address this, Grey et al.^72^ developed and validated HIT–MAP–MALDI–MSI, an open‐source bioinformatics workflow utilising peptide mass fingerprinting and a dual‐scoring system. This workflow computationally assigns peptide and protein annotations to high‐resolution MSI datasets, generating customisable spatial distribution maps. HIT–MAP holds promise for robust peptide and protein annotation and visualisation in diverse health and disease scenarios.^72^

Accurate structural elucidation in MSI remains a challenge; however, there exist some annotation tools or approaches exist that can offer more detailed structural information based not only on accurate mass measurement. For example, Zhu et al.^76^ proposed a knowledge‐guided multi‐layer network (KGMN) to enhance the annotation of unknown metabolites. KGMN integrates a three‐layer network: a knowledge‐based metabolic reaction network (KMRN), a knowledge‐guided MS/MS similarity network and a global peak correlation network. KMRN connects known and unknown metabolites, providing a basis for annotating unknown metabolites. The knowledge‐guided MS/MS similarity network links metabolites on the basis of MS1 mass, retention time, MS/MS similarity and metabolic biotransformation, propagating annotations from known to unknown metabolites. The global peak correlation network identifies different ion forms of the same metabolite, optimising and filtering annotation results. Comparing the KGMN method with manually validated metabolite lists, the correct annotation rate improved from 72.2 to 98.5%, significantly outperforming previous methods. Additionally, Zhu's group established the AllCCS database, which combines comprehensive collision cross section (CCS) data with in situ MS/MS spectral libraries to increase the accuracy of metabolite labelling. CCS is crucial for describing the collision cross‐sectional area of molecules in IM‐MS, reflecting their size, shape and conformation, and is valuable for metabolite identification and annotation. The technique uses an optimised machine learning (ML) model for large‐scale CCS prediction and a five‐step standardisation approach to reconcile experimental CCS results from diverse sources. By addressing CCS variability among instruments and laboratories and providing a reliable method for comprehensive metabolomics research, the combination of AllCCS and MS/MS spectra improves the accuracy and coverage of metabolite annotation.

### 3.2 Advances in biomarker discovery and function visualising

In translational medical research, biomarker identification and visualisation are essential for improving precision medicine, assessing medication efficacy and diagnosing diseases. Metabolomics requires extensive sampling and analysis to predict changes in metabolic pathways and construct metabolic networks. MSI offers an accurate method for visualising the spatiotemporal distribution of bioactive metabolites in spatially resolved metabolomics, aiding in biomarker identification. The advent of computer‐aided diagnostic technologies has significantly enhanced the visualisation of functions in metabolomics. These technologies integrate machine learning (ML) and deep learning (DL) techniques with analytical tools such as principal component analysis (PCA), K‐means clustering and logistic regression. ML and DL techniques enhance the visualisation of metabolite functions and biomarker discovery by automatically learning and uncovering hidden patterns and associations within large, complex MSI datasets. PCA reduces the dimensionality of MSI data, K‐means clustering identifies metabolite clusters, and logistic regression predicts associations between metabolites and diseases. Collectively, these advancements enhance diagnostic and therapeutic evaluations.

To grasp the in vivo behaviour of drug candidates, pinpoint potential therapeutic targets and elucidate the underlying molecular mechanisms, it is critical to examine metabolic alterations following drug administration through the lens of functional metabolites. Considering that no single metabolite can comprehensively depict the intricate changes in metabolism and given MSI's ability to spatially delineate such perturbations, our research team has devised a novel index: the metabolic perturbation score (MPS). This metric, implemented as MPS–MSI, allows pixel‐by‐pixel computation of metabolic disturbance scores, identifying regions with significant alterations. In preclinical studies of paclitaxel derivatives, MPS‐MSI flagged the spleen as a potential site of toxicity and highlighted sensitive tumour microenvironments. The initial results suggest that methionine adenosyltransferase and PE N‐methyltransferase are probable targets. MPS–MSI has emerged as a potent molecular imaging method for assessing drug efficacy and safety in early‐stage development, providing insights for clinical applications by analysing the impact of drugs on various tissues or organs. Additionally, MPS quantitatively describes the extent of a drug's impact on metabolism, aiding in optimising clinical dosing and administration protocols.

Lam et al.^77^ developed MEISTER, a novel MS framework utilising DL for signal reconstruction. This framework generates a 3D whole‐brain atlas at single‐cell resolution through data‐driven multi‐modal alignment. Additionally, they mapped the cell‐specific atlas to tissue imaging data via a dictionary learning algorithm, enabling multi‐scale characterisation. With MEISTER, they conducted region‐specific lipid analyses, identifying unique lipid markers for different brain regions. This approach not only reduces the data acquisition time but also provides a valuable tool for understanding spatial biochemical organisation at the cellular level. MEISTER also aids MSI experiments by integrating high‐throughput MSI and single‐cell mass spectrometry data, enabling multi‐scale biochemical feature analysis. This innovation facilitates the identification of cell‐type‐specific biochemical features and their spatial distribution within tissues. Consequently, MEISTER's multi‐scale biochemical analysis holds promise for clinical applications, such as disease diagnosis, prognosis assessment and the discovery of therapeutic targets. In particular, by offering comprehensive biochemical insights at both the tissue and single‐cell levels, MEISTER enhances the ability to identify biomarkers for various diseases, potentially leading to more accurate diagnostics and personalised treatment strategies. This robust analysis capability positions MEISTER as a powerful tool in clinical translational research, paving the way for advanced therapeutic interventions and improved patient outcomes.

Klingler‐Hoffmann et al.^78^ investigated the clinical potential of applying ML to MALDI–MSI data. By employing a deep neural network‐based approach, they classified cancer diagnoses via tissue microarrays (TMAs) from 302 colorectal cancer patients and 257 endometrial cancer patients. This method distinguished colorectal tumours from normal tissues, achieving an overall accuracy of up to 98% in balanced cross‐validation. Furthermore, the ML approach predicted the presence of lymph node metastasis in endometrial cancer with 80% accuracy, demonstrating the ability of MALDI–MSI to enhance traditional histopathological examinations in cancer diagnostics.^78^

Xiang et al.^79^ explored the use of deep and shallow learning for cancer diagnosis through the analysis of optical and MSI images. Specifically, they applied DL to optical images of FFPE breast tissue data from TMAs and shallow learning to DESI–MSI images of the same samples. Their findings revealed that the combination of DESI–MSI and shallow learning not only distinguishes cancerous from normal tissue but also yielded valuable chemical insights for detailed analysis.^79^ However, identifying the core region of TMAs remains challenging. Furthermore, Peng et al.^80^ introduced mNet, a novel DL framework that employs a bounding box detection algorithm to automate this process, significantly streamlining high‐throughput TMA experiments.

Leveraging the ability of artificial intelligence in tumour identification and categorisation, Maaß et al.^81^ introduced a fully automated system for multi‐modal lung cancer subtype analysis. This system employs DL neural networks on whole‐slide tissue images and MALDI–MSI to detect and segment tumour areas precisely, annotate them accurately and differentiate between adenocarcinoma and squamous cell carcinoma, the most prevalent non‐small cell lung cancer (NSCLC) subtypes. This approach is beneficial for effective treatment strategies.^81^ On the other hand, Jing Juan Xu et al. proposed an innovative strategy called AI‐assisted subcellular MSI (AI‐SMSI) with in situ image segmentation. This method achieves subcellular‐level segmentation and utilises an AI model for in‐depth metabolite analysis in distinct cell regions. Notably, biomarkers that distinguish the drug actions of adriamycin and epirubicin have been identified, paving the way for safer drug development by elucidating the mechanism of isomeric drugs.^82^

## 4 APPLICATION

### 4.1 Disease mechanism and clinical translation

Spatially resolved metabolomics, leveraging the power of MSI, plays a pivotal role in revealing tissue‐specific metabolic patterns, thereby offering invaluable insights into the intricate mechanisms underlying various diseases, including the complex and heterogeneous nature of tumours. Tumour heterogeneity, characterised by diverse cellular subpopulations within a single tumour, poses significant challenges for effective treatment, as these subpopulations often exhibit distinct metabolic profiles and therapeutic responses. By enabling the in situ mapping of metabolic signatures, spatial metabolomics provides a powerful means to dissect this heterogeneity, facilitating a more precise understanding of tumour biology and informing the development of targeted therapies. Beyond oncology, this technology enables a deeper understanding of how metabolic alterations are linked to pathophysiological processes, aiding in the exploration of disease aetiology. The ability to map metabolic signatures in situ bolsters diagnostic precision by revealing true biomarkers associated with certain conditions and aids in predicting disease progression and patient outcomes. By illuminating metabolic pathways dysregulated in disease states, MSI‐guided spatial metabolomics paves the way for the development of targeted, mechanism‐driven therapies, expediting the transition from bench to bedside. It serves as a cornerstone for precision medicine, where tailored interventions are designed based on the basis of individual metabolic profiles, thereby revolutionising clinical translation and patient care strategies.

**FIGURE 4:**
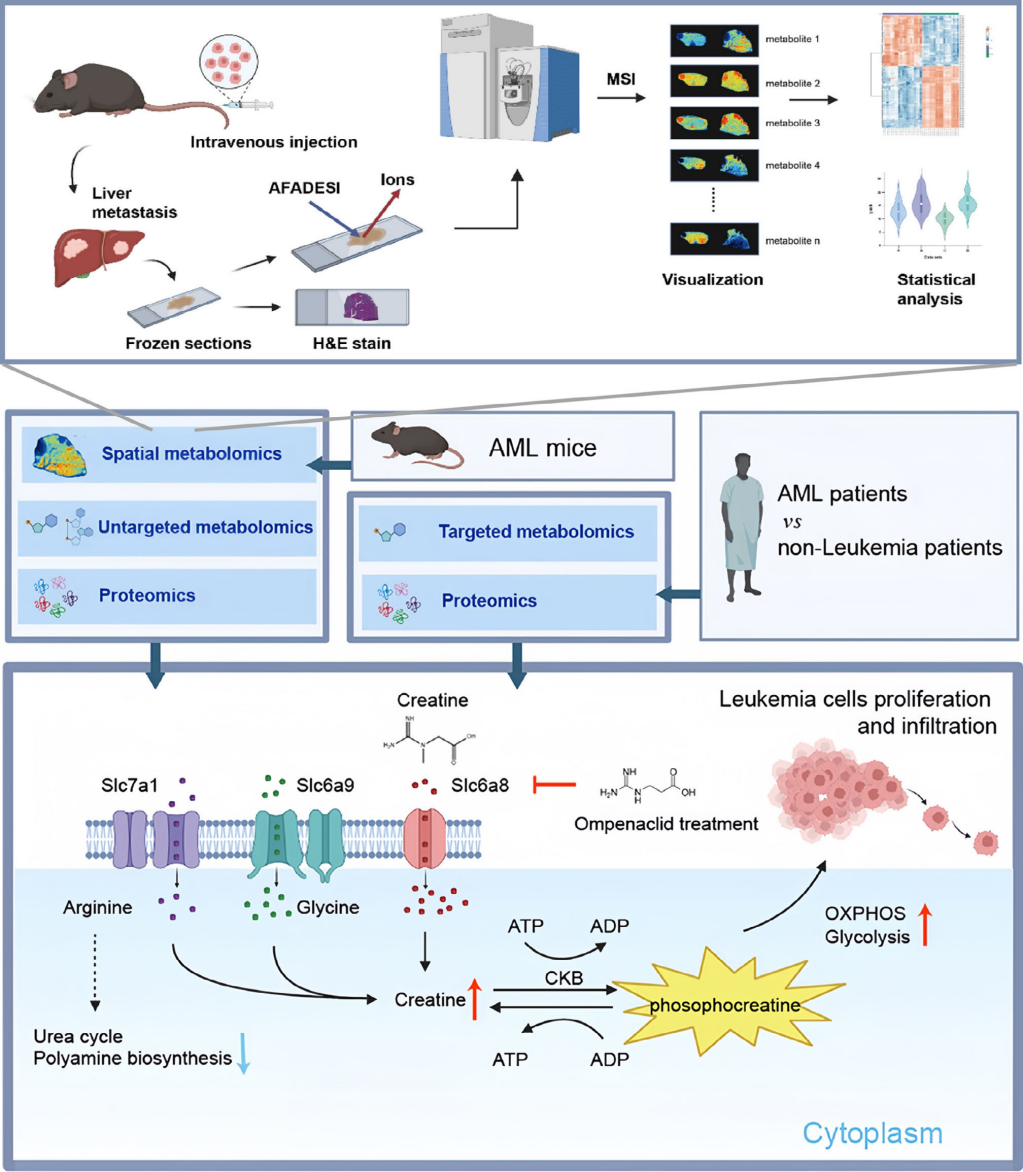
Spatial metabolomic techniques identify metabolic reprogramming features in liver metastases of acute myeloid leukaemia (AML)^85^ ^.^

By visualising the spatial and temporal changes in metabolites at different pathological stages, MSI enables metabolic pathway prediction and disease mechanism prediction. Li et al.^83^ performed the first MALDI‐based lipidomic imaging of arterial cross‐sections in a recurrent, restenotic small animal model after cardiovascular treatment, revealing alterations in biologically active lipids associated with restenotic arterial function at different stages of disease progression.^83^ van Soest et al.^84^ used lipidomics and DESI–MSI to study the effects of carotid arterial endarterectomy in arteries from patients who underwent Lipid profiling of advanced human carotid plaques and plasma obtained from patients undergoing carotid endarterectomy revealed the presence of several molecular species colocalised with relevant disease processes in plaques and the presence of specific lipid species in unstable plaque regions and identified the presence of significant circulating species in plaques as well.^84^ Tongyin et al.^85^ used AFADESI–MSI technology to identify metabolites in the liver metastases of AML mice, mapping over 4000 ions. They discovered that the upregulation of the creatine metabolism pathway was driven by increased expression of creatine synthesis enzymes and the creatine transporter SLC6A8. This accumulation of creatine enhances oxidative phosphorylation and glycolysis in AML cells, boosting ATP production and promoting tumour proliferation. Inhibition of SLC6A8 with ompenaclid countered these effects, suggesting targeting metabolic pathways as a potential AML treatment. This exemplifies how spatial metabolomics can reveal crucial metabolic alterations and potential treatment targets in disease contexts, demonstrating its transformative impact on disease management and therapy development^85^ (Figure 4).

Owing to the complex structure and function of the brain, drug targets and metabolic alterations are difficult to discover using conventional histology. Baykal et al.^86^ used MALDI–MSI coupled with an LC–MS/MS system to study proteomic alterations in the brain tissue of a mouse model of Huntington's disease and identified more than eighty proteins that were significantly different in the control, mild and pathologic phases of the disease. Using AFADESI–MSI, Abliz et al.^87^ identified specific metabolic disorders in rat model of diabetic encephalopathy and identified 19 metabolic pathways.

Spatially resolved metabolomics not only helps in studying disease mechanisms but also plays a crucial role in preventing diseases by identifying and mitigating the effects of harmful compounds through toxicity studies. Aristolochic acid I is a known nephrotoxic drug, and Abliz et al.^88^ identified metabolites associated with nephrotoxicity by directly analysing metabolites in renal tissue sections via a spatially resolved metabolomics approach based on AFADESI–MSI. New insights into the mechanism of aristolochic acid nephrotoxicity are provided.^88^ Perfluorooctanoic acid (PFOA) is a synthetic perfluorinated chemical that is associated with many toxic effects, including liver injury. Stoffels et al.^89^ performed a comprehensive lipidomic analysis of livers from PFOA‐exposed and control mice via LC‒MS/MS, MALDI–MSI and TOF–SIMS. LC–MS/MS was used for quantitative analysis of lipid components in liver samples, identifying significant alterations in over 350 lipids, including PE, phosphatidylcholine (PC) and triacylglycerol. MALDI–MSI revealed the heterogeneous distribution of PFOA and affected lipids within liver tissue. TOF–SIMS provided detailed localisation of PFOA at the cellular level. This multi‐mass spectrometry approach offers a comprehensive understanding of the spatial and quantitative changes in liver lipids due to PFOA exposure, making it a promising tool for toxicological studies. Wan et al.^62^ used AFADESI to map halogenated hydrocarbons and metabolites in zebrafish and discovered that lipids and polyamine/adenosine‐related metabolites are decreased when chlorinated paraffins accumulate in the liver, heart and brain. This underlines the necessity of evaluating the spatial distribution and impacts of pollutants to comprehend toxicity mechanisms more fully.

**FIGURE 5:**
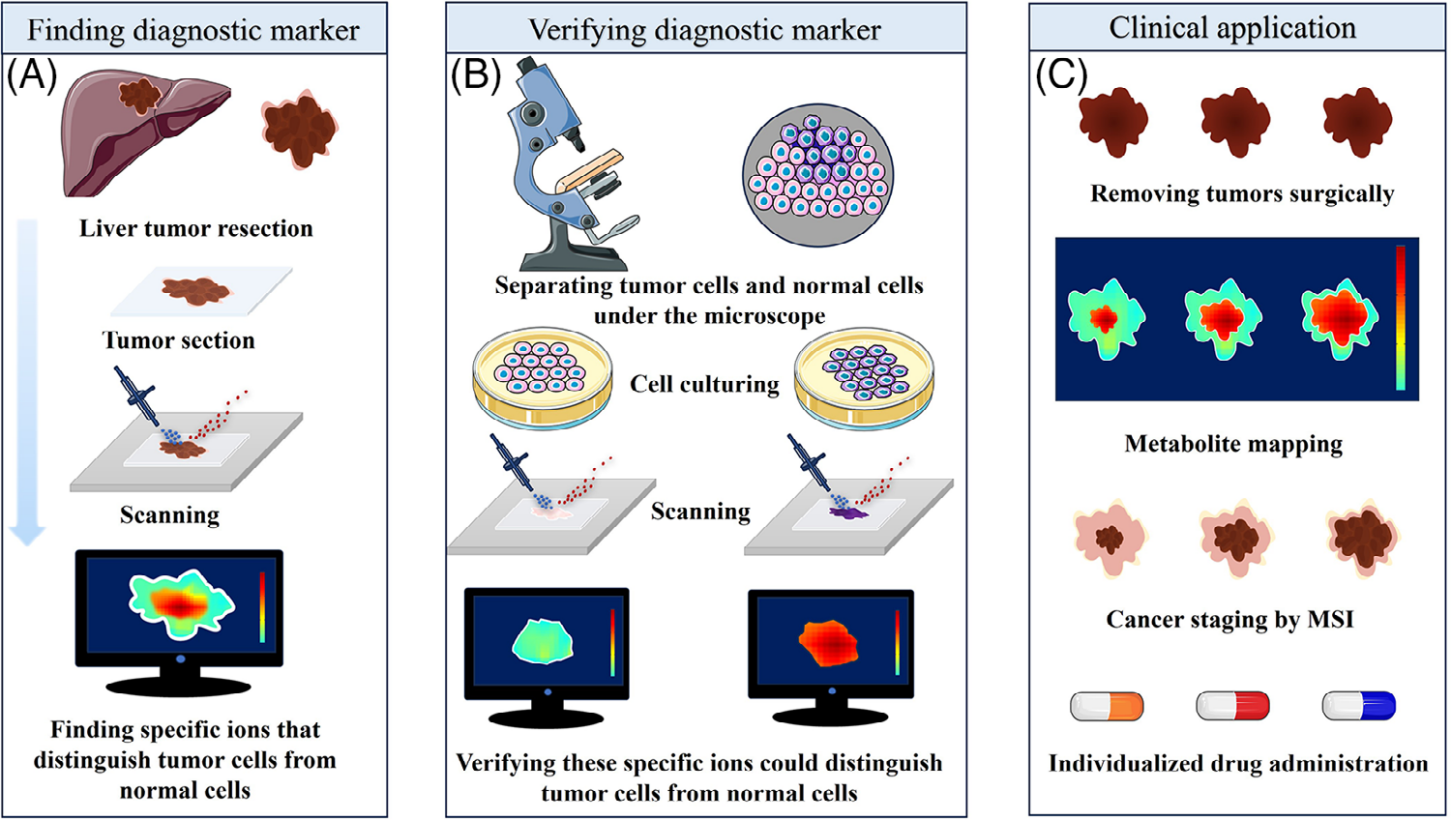
Application of spatially resolved metabolomics in cancer diagnosis and therapy. (A) Frozen sections obtained from clinically resected liver tumour tissue were investigated via MSI technology to visually identify ions that can distinguish between tumour tissues and healthy tissues. (B) The specificity of the ions pinpointed in the previous step is corroborated through microscopic examination, where cultured cancer cells and normal cells, which are maintained separately, are analysed via MSI to confirm their differential expression. (C) Once characterised, these signature ions that distinguish tumours are employed clinically for cancer classification, enabling the adoption of tailored therapeutic regimens and targeted drug delivery strategies.

Clinical translation is the process of applying basic science research results to clinical practice, and MSI has great potential for clinical diagnostics, including the discovery and rapid detection of tumour markers, the differentiation of cancerous tissues from normal tissues for the assessment of surgical resection margins (Figure 5), the staging of various diseases with distinctive features, and spatially resolved metabolomics, which is expected to contribute to the development of translational medicine.

Moreover, the exploration of spatial heterogeneity within tumour tissues through MSI has further advanced our understanding of disease complexity and contributed to more precise clinical diagnostics. The exploration of spatial heterogeneity within tumour tissues through MSI has significantly advanced our understanding of disease complexity, enhancing the precision of clinical diagnostics. Spatially resolved metabolomics via MSI has become a vital tool for revealing tumour heterogeneity by visualising and analysing the distributions of lipids, metabolites and proteins within tumour tissues while maintaining tissue morphology. Studies by researchers such as Zeper Abliz, Axel Walch and Per Malmberg have identified significant disruptions in key metabolic pathways, including glutamine metabolism, fatty acid synthesis and amino acid metabolism, which not only distinguish tumour tissues from normal tissues but also predict tumour progression.

For example, Abliz and colleagues^90^ used MSI to study multi‐cellular tumour spheroids (MCTSs) derived from KYSE‐30 oesophageal cancer cells, revealing heterogeneous distributions of critical metabolites that highlight metabolic diversity within tumours. Similarly, Walch's team^91^ applied MALDI–FTICR–MSI to adrenocortical carcinoma (ACC) samples, identifying 12 distinct metabolic subgroups, with greater heterogeneity correlated with increased malignancy and poorer prognosis. Per Malmberg's research using TOF–SIMS on glioblastoma (GBM) samples revealed heterogeneous lipid distributions, indicating regional differences in tumour biology.^92^

Further innovations include the work of Gao and colleagues,^93^ who developed a protocol that integrates SMALDI–MSI to distinguish breast cancer from normal tissues on the basis of lipid distribution while preserving structural integrity. The integration of MSI with other imaging modalities, such as immunohistochemistry (IHC) and spatial transcriptomics, as demonstrated by Walch's research^94^ in gastric cancer, has led to the identification of distinct metabolic subtypes with significant implications for prognosis and personalised therapy. Additionally, Heeren's team^95^ employed MALDI–IHC for multiplexed imaging of breast cancer tissues and detected protein markers at single‐cell resolution, which further refined the understanding of tumour heterogeneity. Research by our group revealed the complexity of metabolic reprogramming and cell interactions within tumours via spatial metabolomics techniques such as AFADESI–MSI and MALDI–MSI.^96^ Raymond's^97^ exploration of β‐catenin mutated hepatocellular adenomas through spatial metabolomics highlighted the heterogeneity at tumour margins, highlighting the importance of this approach in understanding liver tumour pathogenesis.

Finally, Cai's research^98^ on 3D MCTSs demonstrated that these models replicate the lipid metabolism and distribution patterns observed in solid tumours, particularly in necrotic regions, validating MCTSs as valuable in vitro models for studying tumour heterogeneity. Collectively, these advancements in spatial metabolomics are deepening our insights into tumour biology, facilitating the identification of potential biomarkers, and informing therapeutic strategies tailored to the molecular characteristics of individual tumours.^98^

One of the main obstacles preventing the clinical intraoperative diagnostic use of MSI is its inability to facilitate rapid sample processing and data acquisition. To overcome this, Basu et al.^99^ introduced a swift MALDI–MSI method that completes both sample preparation and analysis in under 5 min, addressing the major obstacle of rapid sample processing and data acquisition in clinical intraoperative diagnostics. The workflow involves quickly cryosectioning surgical samples for HE staining and rapid digital imaging, whereas matrix‐precoated indium tin oxide (ITO) slides are directly analysed via MALDI mass spectrometry. Two diagnostic approaches are used: the pathologist‐guided mode, which fuses HE‐stained images with ion images, and the AI‐guided mode, which uses ML models for data analysis. This method, which integrates seamlessly with the clinical frozen section workflow, significantly reduces the diagnostic time. Enhancements such as matrix‐precoated ITO slides eliminate the matrix spraying step, a high‐frequency 10 kHz laser, and optimised scanning mode reduce the data acquisition time by 10−20 times, improving the speed and efficiency of studying clear cell renal cell carcinoma (ccRCC). Rapid and precise discrimination between cancerous and healthy tissue is crucial, especially for ccRCC, the most prevalent and lethal renal cancer subtype. Brooks’ study^100^ utilised DESI–MSI for diagnosing and prognosticating ccRCC. This method rapidly and accurately detected metabolites in both healthy and ccRCC tissues, improving the scanning efficiency and identification accuracy to 82% for normal tissues and 88% for ccRCC tissues. Advancements in this technology could enhance rapid intraoperative surgical margin assessments, potentially improving clinical outcomes.^100^ Likewise, Walch et al.^101^ improved AI‐based diagnosis and tumour subtype classification via the combination of MALDI–MSI and computer‐aided image analysis.

Rapid evaporative ionisation mass spectrometry (REIMS) of electrosurgical vapours allows real‐time classification of healthy and tumour tissues in various surgical procedures. However, for oral squamous cell carcinoma, REIMS must detect small numbers of tumour cells. Heeren et al.^102^ assessed REIMS sensitivity to determine the minimum detectable tumour cell count in oral cancer surgery. Additionally, complementary DESI–MSI was employed to map tissue heterogeneity in six oral sections, corroborating the REIMS findings. The assessment of tissue heterogeneity via DESI–MSI and REIMS sensitivity to cell mixtures revealed sensitive metabolic profiles for in vivo tissue identification during oral cancer surgery.^102^

Ovchinnikova's team^103^ utilised DL methods to align features extracted from H&E images with MSI data, achieving approximately 80% accuracy in predicting prostate cancer. This method holds promise for rapid cancer diagnosis in the future.^103^ To improve diagnostic accuracy, our team used spatially resolved metabolomics analysis based on AFADESI–MSI to establish a molecular diagnostic strategy for distinguishing four pathological types of thyroid tumours. Three sets of metabolic biomarkers for visual differentiation of benign follicular adenomas and differentiated thyroid carcinomas were identified via microregion feature extraction and metabolomics analysis, and a diagnostic model based on the metabolic profiles of 65 thyroid nodules supported the automatic prediction of tumour lesions. When a test set of 12 independent samples was used, the model achieved 83.3% accuracy. This diagnostic strategy provides a new approach to in situ pathology using small molecule biomarkers and provides a model for the diagnosis of clinically indeterminate thyroid tumours.^104^

Rapid detection of tumour markers from frozen sections can facilitate intraoperative histopathological studies and reduce the need for secondary surgery. Banerjee et al.^105^ deciphered diacylglycerol as a potent biomarker for the diagnosis of breast cancer via DESI–MSI of lumpectomy specimens. Metabolic pathway analysis revealed that increased catabolism of PC in breast cancer contributes to diacylglycerol overexpression. These results provide an opportunity to map diacylglycerol signalling in breast cancer in the context of new therapeutic and diagnostic developments, including intraoperative assessment of breast cancer margin status.^105^

### 4.2 Drug research and development

Spatially resolved metabolomics, with its unique capacity for intricate visualisation and comprehensive analysis, revolutionises our understanding of pharmacokinetics by pinpointing the precise spatiotemporal dynamics of exogenous drugs and their metabolites, alongside a myriad of endogenous metabolites. This approach is instrumental in deciphering the intricate mechanisms governing drug metabolism transformations, offering unprecedented insights into how drug candidates navigate and interact within biological systems. Through meticulous comparison of metabolite profiles before and after drug intervention, the efficacy and toxicity profiles of compounds were determined meticulously, ensuring a thorough appraisal of pharmaceutical interventions.

**FIGURE 6:**
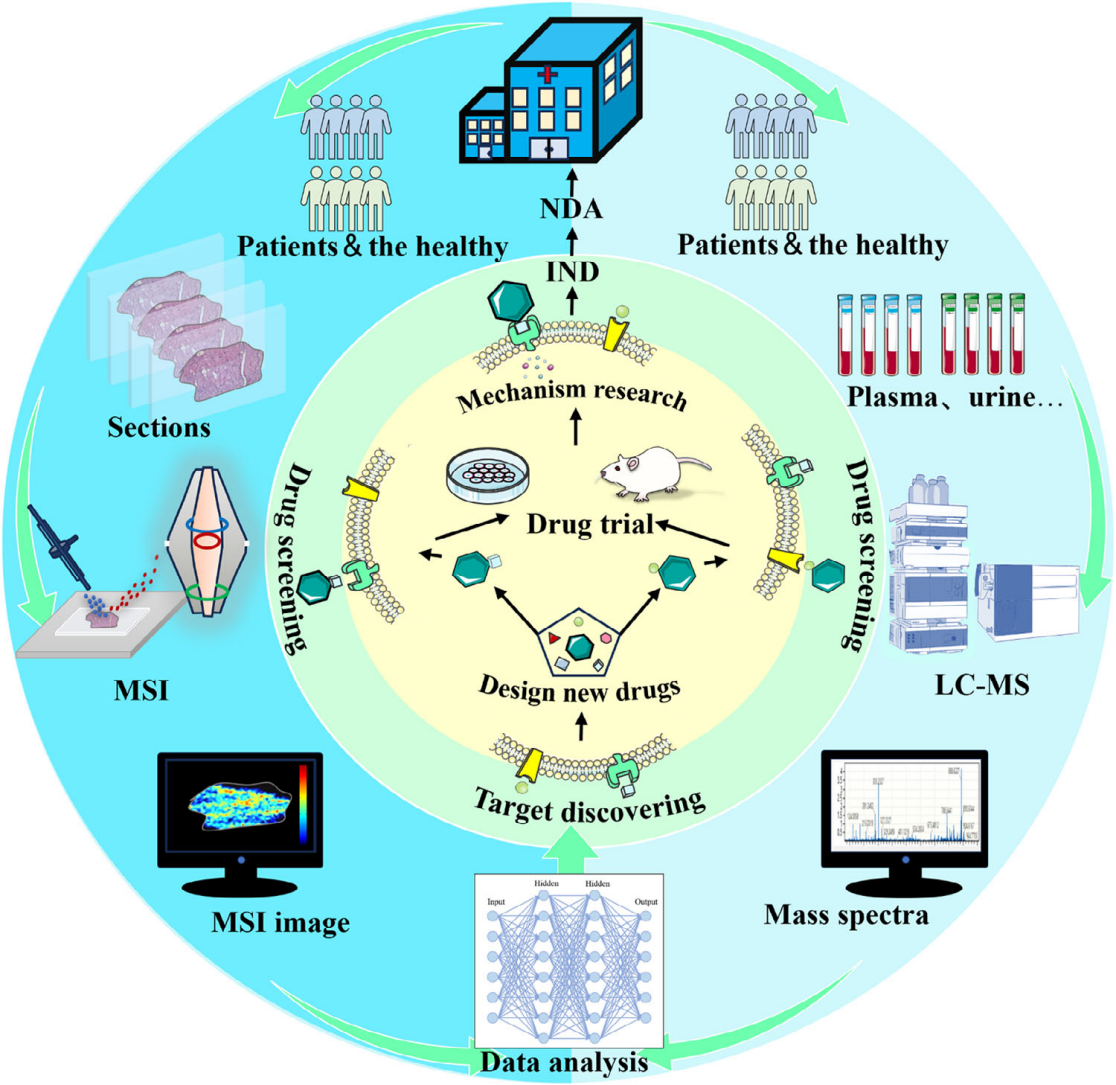
LC‒MS‐based metabolomics and spatially resolved metabolomics in the new drug development process. (A) Discover potential drug targets by analysing clinical samples; (B) conduct drug design based on potential drug targets; (C) synthesise the new drug and conduct preclinical trials; (D) conduct drug mechanism research based on the results of the drug trials; (E) IND filing: conduct pre‐market human trial declaration and research; (F) NDA declaration: clinical trials of new drugs are completed, and the declaration of registration and listing.

The power of the technique lies in visually mapping the dispersion of drugs and bioactive metabolites, thereby unmasking the sites of metabolic activity and critically informing the validation and discovery of therapeutic targets. This granular view not only strengthens our grasp of existing drug mechanisms but also illuminates previously unseen avenues for optimising drug performance and reducing unwanted toxicities. By illuminating these metabolic landscapes, spatially resolved metabolomics charts a course for the future of new drug development, guiding researchers toward innovations that promise enhanced therapeutic benefits while mitigating adverse effects, thereby advancing the frontier of precision medicine (Figure 6).

Dannhorn et al.^106^ determined the distribution and relative abundance of four benchmark compounds in intestinal segments via DESI–MSI and MALDI–MSI. The drug distribution in different intestinal histological compartments was determined by MALDI–MSI at a high spatial resolution of 10 µm, which helps identify the absorbed and tissue‐bound fractions of the drug.^106^ Kwon et al.^107^ utilised MALDI–MSI to investigate the distribution of erlotinib in drug‐resistant and drug‐sensitive NSCLC mouse xenograft models. The amount of erlotinib in tumours is much lower than that in the liver and kidneys, leading to the identification of a novel resistance mechanism in NSCLC.^107^ Müllertz et al.^108^ used MALDI–MSI to visualise the distribution and spatial location of a model prodrug (fenofibrate) via the gastrointestinal tract (GIT) in rats and detected that fenofibrate was hydrolysed to the already gastric active drug fenofibric acid and confirmed the presence of lysophosphatidylcholine (lyso‐PC) and taurocholate in the lumen of the small intestine, validating the activation of the active drug and advancing the study of the mechanism of action of the drug in the GIT. Using AFADESI–MSI, Jin et al.^109^ visualised the spatial distribution of endogenous metabolites within lung tissue. Furthermore, they delved into the putative metabolic mechanism underlying the therapeutic effects of Prismatomeris connate ethyl acetate extract (HG‐2) on pulmonary fibrosis improvement, leveraging spatially resolved metabolomics.^109^ In addition, our team proposed a spatiotemporally resolved metabolomics and isotope tracing strategy. This strategy was used to discover potential targets of the anti‐insomnia drug candidate YZG‐331 and provided a viable way to link metabolic biomarkers to protein targets in metabolic pathways.^110^

MSI is further capable of distinguishing the spatial distribution of the intact parent drug, its various metabolites and even endogenous compounds, thereby offering valuable insights into pharmacokinetic profiles. Setou et al.^38^ visualised the distribution of APAP and APAP–CYS in the kidney at different metabolic time points without the need for derivatisation by AP‐MALDI, identified a new metabolite tentatively designated APAP–BS and revealed for the first time the differential distribution of the three. Rebouta et al.^111^ used DESI–MSI to study the spatial distribution of tolcapone in rat liver and brain coronal and striatal sections after a single oral dose of 100 mg/kg of tolcapone, a brain‐permeable compound. Tolcapone is uniformly distributed in liver tissue sections and specifically distributed in the brain, which helps to reveal its intracerebral pharmacodynamics.^111^ Our team created a temporospatial pharmacometabolomic approach to clearly depict the pharmacokinetics of the prototype olanzapine (OLZ) and its metabolite 2‐hydroxymethyl OLZ in several microregions of the rat brain. The evaluate the microregional impact of OLZ on brain tissue was further evaluated, thereby revealing its efficacy in delineating the intricate microregional pharmacokinetics and pharmacodynamics of central nervous system medications that successfully traverse the blood‒brain barrier.^112^

Abliz's team^113^ investigated tissue‐specific metabolic alterations in the kidneys of high‐fat diet‐fed and streptozotocin‐treated diabetic nephropathy (DN) rats and the therapeutic efficacy of the potential anti‐diabetic drug astragaloside IV on DN on the basis of AFADESI and MALDI–MSI; a variety of metabolites were identified, and multiple metabolic disturbances were found to occur in a region‐specific manner. These region‐specific metabolic disturbances were ameliorated by repeated oral administration of astragaloside IV for 12 weeks, demonstrating the therapeutic efficacy of astragaloside IV for DN.^113^

Visualisation of drug metabolism facilitates formulation optimisation and new drug development. In skin penetration studies of drugs, qualitative skin distribution analysis of endogenous molecules and the drug molecule tofacitinib via MALDI–MSI and quantitative analysis of the amount of tofacitinib in the epidermis allows for the exploration of the rate of release of the drug in different cream formulations as well as the rate of skin retention, which can be used for formulation optimisation.^114^ The localisation of the commonly used anti‐inflammatory drug diclofenac and its metabolites in mouse kidney and liver tissues was examined via nano‐DESI–MSI. The observation that diclofenac acyl glucosinolates and hydroxy‐diclofenac are localised in the inner medulla and cortex of the kidney, respectively, is important for subsequent studies on how to mitigate side effects such as gastrointestinal toxicity and hepatic failure that may result from their metabolism.^115^

Through MSI‐based spatially resolved metabolomics, new prospects for targeted anti‐cancer drug development have been identified, informing the design and validation of a novel class of drug candidates with high specificity and reduced toxicity. Using spatially resolved metabolomics, we identified a broadly upregulated choline metabolic phenotype in tumours from heterogenous clinical tumour tissues and various tumour models, and then devised a choline‐targeted small molecule drug conjugate strategy. This approach enhances tumour targeting, maintaining therapeutic efficacy while reducing paclitaxel‐induced toxicity, thus illustrating the potential for creating low‐toxicity, highly efficacious anti‐tumour agents. This breakthrough presents a promising avenue for precision‐targeted chemotherapy in lung, pancreatic and other cancers.^116^

## 5 CONCLUSION AND PROSPECTS

MSI has powered spatially resolved metabolomics as a fundamental bioanalytical tool in life science, overcoming the shortcomings of LC‒MS‐based metabolomics—the loss of vital spatial information within heterogeneous tissue samples. This technology excels in unravelling the mechanism of diseases, discovering biomarkers and meticulously evaluating drug toxicity and treatment outcomes. It achieves this goal by transforming the cryptic world of metabolites and their intricate networks into vivid, spatially explicit images, thereby rendering biological insights tangible and actionable. However, the field still faces significant technical challenges, including issues related to sample pretreatment, such as the risk of sample degradation and the trade‐off between spatial resolution and sample thickness. In addition to these challenges, MSI still face a balance between spatial resolution and sensitivity. Enhancing spatial resolution often results in reduced signal intensity per pixel, which can compromise sensitivity. Moreover, the complexity of quantitative analysis in MSI, compared with that in LC‒MS, poses a significant hurdle, primarily due to matrix effects, ion suppression and signal inconsistency. Addressing these challenges will require the development of new laser or ion beam technologies and improvements in sample preparation methods, as well as the integration of ML to increase quantification accuracy. Moreover, the efficient extraction and accurate representation of metabolites from tissue samples pose considerable difficulties.

This review underscores MSI‐driven spatial metabolomics as a formidable approach for cutting‐edge bioanalysis, highlighting pivotal advancements on two fronts: (1) innovations in preanalytical processing techniques and MSI hardware that increase sensitivity, comprehensiveness and precision in metabolite capture and (2) advancements in functional visualisation methodologies, refining the accuracy of metabolite characterisation and annotation. These enhancements collectively increase the capacity to discern intricate metabolic patterns and their variations in health and disease. Despite these advancements, MSI's data processing and analysis remain significant challenges because of the complexity and high dimensionality of the data it generated. Future research should focus on developing more efficient data processing tools, advanced algorithms for noise reduction and comprehensive data sharing platforms. These developments are essential for managing the enormous datasets produced and facilitating collaboration among researchers.

MSI has significant potential as a critical tool in spatially resolved metabolomics, leading to notable advancements in metabolite quantification and high‐resolution imaging through quantitative MSI. The ‘chemical quant array’ method enhances quantification precision with isotope‐labelled standards, automatic pixel removal and internal standard normalisation.^117^ This improves analysis efficiency by reducing calibration standard occupancy in MALDI–MSI and enables multi‐metabolite quantification, thus increasing clinical applicability. The method addresses MALDI–MSI's quantitative challenges, supporting clinical and translational research, such as tumour metabolite monitoring. The use of quantitative MSI promises better accuracy, reduced analysis time and broader applications in clinical medicine. Nonetheless, the preparation and handling of samples in MSI continue to be a significant technical hurdle. The need to preserve spatial integrity during preparation, coupled with the challenges of ensuring consistent sample thickness and uniform matrix application, can lead to variability in results. Future research should prioritise the development of automated sample preparation techniques to minimise human error and enhance reproducibility.

The review illustrates the immense potential of spatially resolved metabolomics in bridging the gap between the bench and the bedside. It outlines how this technology can facilitate the translation of biomedical discoveries into precision medicine strategies and streamline the development of novel therapeutics, thereby underscoring its pivotal role in advancing healthcare and pharmaceutical innovation. Additionally, the diverse and complex nature of MSI technologies—ranging from MALDI–MSI to DESI–MSI—presents another layer of challenge in optimising experimental conditions across different platforms. Future research efforts should aim to systematically evaluate the strengths and limitations of various MSI technologies and explore hybrid approaches that combine the best features of each method.

A forefront area of advancement in spatially resolved metabolomics is the progression toward achieving cellular and even subcellular‐level spatial resolution, pushing the boundaries of analytical precision. Sweedler et al.^118^ accomplished ultrahigh‐resolution imaging of individual hippocampal cells in animals via MALDI‐21 T FTICR, which reached the single‐cell and subcellular levels. Zare et al.^119^ employed a microlens fibre coupled with various MSI techniques to achieve subcellular resolution. Hang et al.^120^, ^121^ utilised microlens fibre laser desorption MSI, achieving a resolution of 300 nm and visualising the subcellular distribution of small molecule drugs and nanomaterials. However, with advancements in spatial resolution, the complexity of addressing matrix effects and ion suppression at these finer scales becomes more pronounced. Future research should focus on developing more uniform matrix application techniques and investigating matrix‐independent imaging methods to mitigate these issues.

With the refinement of methodologies and significant strides in MSI technology, spatially resolved metabolomics has attained unprecedented levels of spatial and mass resolution, efficient data acquisition rates and an expanded scope of metabolite coverage. A notable trend is the development of ML‐powered approaches, which have accelerated and enhanced the accuracy of annotating large MSI datasets. This progression ushers in an era of automated data processing, enabling profound data exploration and the potential for AI‐driven disease prognosis. Examples include automated lung cancer subtype analysis via a DL neural network by Maaß et al.,^81^ the AI‐SMSI strategy for automated regional metabolite analysis by Xu et al.,^82^ and prostate cancer prediction via DL techniques by Ovchinnikova et al.^103^

Spatially resolved metabolomics in conjunction with other histological techniques of spatial multi‐omics has the ability to probe upstream and downstream relationships and changes from genes to metabolic processes and will be a major focus of research in the future. Heiland et al.^122^ combined spatially resolved transcriptomics, spatially resolved metabolomics and proteomics to decipher the bidirectional tumour‒host interdependence in GBMs. Similarly, our group integrated spatially resolved metabolomics with lipidomics and spatial transcriptomics to hierarchically visualise gastric cancer samples, revealing cell‐specific metabolism and interactions in gastric cancer.^96^ Humphreys et al.^123^ combined simultaneous high‐throughput single‐cell ATAC/RNA sequencing and spatially resolved metabolomics to analyse different human kidney samples, revealing and highlighting their anatomical heterogeneity and unique metabolomic profiles. Moving forward, it is essential to continue integrating MSI with other multi‐omics approaches while also addressing the inherent challenges of data integration, spatial resolution and sensitivity. By optimising these technologies and focusing on standardisation, MSI can play an increasingly central role in comprehensive spatial multi‐omics studies.

## AUTHOR CONTRIBUTIONS

**Xinyue Min**: Writing—Original Draft and Editing. **Yiran Zhao; Meng Yu; Wenchao Zhang**: Design the figures. **Xinyi Jiang; Kaijing Guo; Xiangyi Wang**: Conceptualisation; data Curation. **Jianpeng Huang; Tong Li**: methodology; supervision. **Lixin Sun and Jiuming He**: Funding Acquisition; writing—review. All the authors have read and approved the final version of the manuscript and agree with the order of presentation of the authors.

## ETHICS STATEMENT

These issues are not applicable for this review.
